# Amelioration of radiation-induced skin injury by tetrahydrobiopterin: preclinical study and phase II trial

**DOI:** 10.1186/s43556-025-00246-x

**Published:** 2025-01-26

**Authors:** Kemin Li, Bin Song, Rutie Yin, Shuyu Zhang

**Affiliations:** 1 The Department of Obstetrics and Gynecology, West China Second University Hospital of Sichuan University, Chengdu, 610041 China; 2 Laboratory of Radiation Medicine, NHC Key Laboratory of Nuclear Technology Medical Transformation, School of Basic Medical Sciences & Forensic Medicine, Sichuan University, Chengdu, 610041 China

**Dear editor,**

Radiation-induced skin injury (RISI) poses a notable challenge in both radiotherapy and radiation-related incidents [1]. Unlike standard wounds, this type of injury is characterized by its hindered healing process and a higher likelihood of recurrence. In the absence of protective measures, the incidence of RISI can exceed 90%. While mild RISI may heal spontaneously without treatment, severe RISI can lead to necrotic wounds and ulceration that complicate healing, ultimately necessitating the cessation of radiotherapy for affected patients [2]. Tetrahydrobiopterin (BH4) serves as a vital cofactor for nitric oxide synthase (NOS) as well as aromatic amino acid hydroxylases. The balance of BH4 is essential for the proper functioning of NOS, which directly influences the production of nitric oxide (NO) and reactive oxygen species. In environments with oxidative stress, including exposure to ionizing radiation, the availability of BH4 can diminish due to oxidation. This reduction can result in the uncoupling of NOS and the generation of extremely reactive free radicals, which can impact the radiosensitivity of cells. BH4 plays an important role in radiotherapy, not only affecting cellular radiosensitivity, but also serving as a potential target for improving the efficacy of radiotherapy and reducing normal tissue damage. Our previous reports have demonstrated the effects of radiation on BH4, leading to an increase in reactive oxygen species (ROS) cascade [3]. We have previously reported that GTP cyclohydrolase 1 (GCH1) mitigates the decrease in cellular BH4 levels induced by radiation, while also inhibiting subsequent reactive oxygen species (ROS) production. This highlights the essential function of BH4 and its synthesizing enzyme, GCH1, in the context of radiation-induced ROS and the associated skin damage [3]. The synthesis rate of BH4 through the de novo pathway is contingent upon the function of its rate-limiting enzyme. Inhibition of GCH1 in vivo leads to oxidative stress in the vasculature and a decrease in leukocyte counts following radiation exposure [4]. In this study, our objective is to investigate the biological effect of BH4 in the progression of radiogenic skin injury.

To begin, we initially assessed the impact of BH4 on the radiosensitivity of skin cells by treating them with various concentrations of BH4. The results indicated that treatment of irradiated skin cells with low concentrations of BH4 not only promoted the proliferation of HaCaT cells but also inhibited radiation-induced apoptosis in these cells (Fig. S1a). Furthermore, we observed a significant reduction in the levels of apoptosis-related proteins, such as Cleaved PARP, following the treatment of irradiated skin cells with low concentrations of BH4 (Fig. S1b). These findings demonstrate that BH4 offers protection to embryonic skin fibroblast cells against radiation-induced damage.

Additionally, we elucidated the effect of *GCH1* deletion on mouse skin radiosensitivity in *GCH1*^*fl/fl*^; *Krt14* (*GCH1* wild-type) and *GCH1*^*fl/fl*^; *Krt14*-Cre^−/−^ (*GCH1* cKO) mice (Fig. S1c). After subjecting the mice to an electron beam radiation dose of 35 Gy, we assessed skin injuries across various groups using a semiquantitative score ranging from 1 to 5. Compared with *GCH1* wild-type mice, the deletion of *GCH1* resulted in impaired skin structure and development in the irradiated subjects. Additionally, we also observed that *GCH1* cKO mice exhibited more pronounced cutaneous injury following radiation exposure (Fig. S1d). These findings indicated that the loss of *GCH1* aggravates radiogenic skin injury, indicating a critical role of BH4 catabolism in this disease.

BH4 (Sapropterin dihydrochloride) is a medication clinically utilized for the treatment of hyperphenylalaninemia. It was first approved by the United States Food and Drug Administration (US FDA) in December 2007 and by the European Food and Drug Administration (EU FDA) in December 2008, subsequently obtaining orphan drug status. It was later approved by the China Food and Drug Administration (SFDA). However, the role of BH4 in radiogenic skin injury diseases and the results of clinical trials have not been documented. In this study, we investigated the safety and preliminary effectiveness and safety of BH4 in patients with vulvar cancer receiving radiotherapy (registration No. NCT05114226). Approval for the study was granted by the Institutional Review Board and Ethical Committees at West China Second University Hospital of Sichuan University (Chengdu, China). Patients were administered BH4 topically (the orphan drug Kuvan®; 1 mg/mL) or were placed under observation. Prophylactic application of BH4 to the irradiated skin area commenced prior to the first session of radiation therapy. Sterile gauze saturated with the BH4 solution was applied to the skin in the radiotherapy area for 15 min, three times daily, continuing for three months post-treatment. The extent of skin damage and the level of pain were evaluated weekly using the RTOG acute radiation injury grading standards and a numeric rating scale for pain [5]. The occurrence of RISI is illustrated in Fig. 1a.

**Fig. 1:**
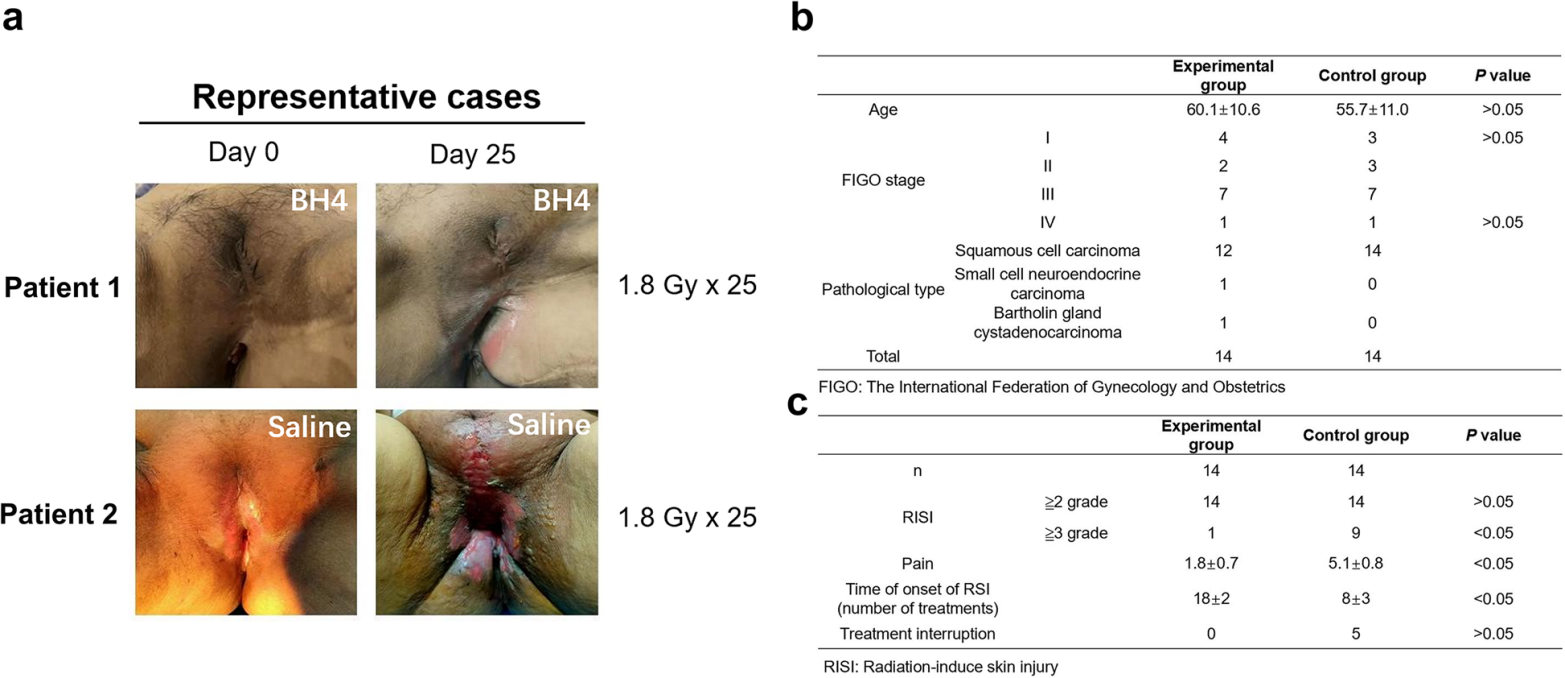
BH4 attenuates acute radiotherapy-induced skin injury of cancer patients. **a** Acute skin injury before and after radiotherapy in the experimental group: Following 1.8 X 25 Gy treatment, the experimental group experienced RTOG grade II skin damage, characterized by alopecia, moderate erythema, moderate edema, and a slight burning sensation. The control group exhibited RTOG grade IV skin damage after the same treatment, which manifested itself as wet desquamation, blisters, and extensive ulceration and necrosis of the skin. The pain resulting from these injuries significantly impacted the participants' ability to walk, sleep, and engage in self-care. **b** and **c** Information on basic characteristics of clinical patient treatment and pain scores. The results are shown as mean ± SEM. *P < 0.05 and ** *P* < 0.01, compared with the control group

A total of 28 patients were enrolled, with 14 patients in each group. The basic characteristics of the participants are presented in Fig. 1b and 1c. The incidence rate of RTOG grade III and above radiation-induced skin injury in the experimental group was 7.1% (1/14), which was significantly lower than the 64.3% (9/14) observed in the control group (odds ratio (OR): 0.04 (0.00, 0.43), *P* < 0.05). Additionally, the treatment pain score for patients in the experimental group was 1.8 ± 0.7 points, significantly lower than the control group’s score of 5.1 ± 0.8 points (*P* < 0.05). The occurrence time of radiogenic skin injury in the experimental group was 18 ± 2 days, significantly delayed compared to the control group’s 8 ± 3 days, (*P* < 0.05). Notably, no patients in the experimental group discontinued treatment due to skin damage, whereas 5 patients in the control group did. These results suggest that BH4 may be effective in the treatment of radiogenic skin injury.

Taken together, this study reveals that GCH1 loss aggravates radiogenic skin injury. Topical administration of BH4 protects against radiogenic skin injury in patients undergoing radiotherapy.

## Supplementary Information

Supplementary Material 1.

Supplementary Material 2.

Supplementary Material 3.

## Data Availability

Data are available upon request to the corresponding authors.

## References

[1] DiCarlo AL, Bandremer AC, Hollingsworth BA, Kasim S, Laniyonu A, Todd NF, et al. Rios CI: cutaneous radiation injuries: models, assessment and treatments. Radiat Res 2020, 194(3):315–344.10.1667/RADE-20-00120.1. 10.1667/RADE-20-00120.1 PMC7525796 32857831

[2] Ryan JL: Ionizing radiation: the good, the bad, and the ugly. J Invest Dermatol 2012, 132(3 Pt 2):985–993. 10.1038/jid.2011.411. 10.1038/jid.2011.411 PMC3779131 22217743

[3] Xue J, Yu C, Sheng W, Zhu W, Luo J, Zhang Q, et al. The Nrf2/GCH1/BH4 axis ameliorates radiation-induced skin injury by modulating the ROS cascade. J Invest Dermatol 2017,137(10):2059–2068. 10.1016/j.jid.2017.05.019. 10.1016/j.jid.2017.05.019 28596000

[4] Berbée M, Fu Q, Kumar KS, Hauer-Jensen M: Novel strategies to ameliorate radiation injury: a possible role for tetrahydrobiopterin. Curr Drug Targets. 2010, 11(11):1366–1374. 10.2174/1389450111009011366. 10.2174/1389450111009011366 PMC3311028 20583982

[5] Cox JD, Stetz J, Pajak TF: Toxicity criteria of the Radiation Therapy Oncology Group (RTOG) and the European Organization for Research and Treatment of Cancer (EORTC). J Radiat Oncol Biol Phys1995,31(5):1341–1346. https:// 10.1016/0360-3016(95)00060-C. 10.1016/0360-3016(95)00060-C 7713792

