# Supplementary material for: Amelioration of radiation-induced skin injury by tetrahydrobiopterin: preclinical study and phase II trial
Source: Mol Biomed. 2025 Jan 26;6:5. doi: 10.1186/s43556-025-00246-x (PMC11762022; doi:10.1186/s43556-025-00246-x)
Supplement: Supplementary file 1 — Supplementary Material 1. [file 43556_2025_246_MOESM1_ESM.docx]

# Amelioration of radiation-induced skin injury by tetrahydrobiopterin: preclinical study and phase II trial

Kemin Li^1^**^#^**, Bin Song^2^ **^#^**, Rutie Yin^1*^, Shuyu Zhang^2*^

1.The Department of Obstetrics and Gynecology, West China Second University Hospital of Sichuan University, Chengdu 610041, China.

2. Laboratory of Radiation Medicine, NHC Key Laboratory of Nuclear Technology Medical Transformation, West China School of Basic Medical Sciences & Forensic Medicine, Sichuan University, Chengdu 610041, China.

#The first two authors contributed equally to this work.

***Corresponding authors:**

R Yin, The Department of Obstetrics and Gynecology, of West China Second University Hospital of Sichuan University, Chengdu 610041, China. Tel/Fax: +8628-85502429; E-mail: yrtt2013 @163.com.

S Zhang, Laboratory of Radiation Medicine, West China Second University Hospital, Sichuan University, Chengdu 610041, China. Tel/Fax: +8628-85502429; E-mail address: zhang.shuyu@hotmail.com or zhangshuyu@scu.edu.cn.

**Supplementary Materials and Methods**

**Matericals**

**Patient:eligibility criteria:**

1. Age and Gender: Patients must be female and over 18 years old.

2. Disease Diagnosis and Condition:

(1) Patients with vulvar cancer diagnosed pathologically and requiring adjuvant radiotherapy after surgery;

(2) Patients with vulvar cancer undergoing radical chemoradiotherapy (including those with unresectable locally advanced tumors, such as some stage I patients with a tumor diameter > 4 cm or tumors invading the vagina, urethra, or anus, and early - stage patients who cannot undergo surgery due to the possibility of severe complications or severe comorbidities).

3. Performance Status: ECOG PS score of 0-1.

4. Expected Survival: Expected survival period is more than 12 months.

5.Endpoints of the Study:

(1) Degree of skin injury at the end of radiotherapy (assessed according to RTOG criteria).

(2) Time of initial radiation-induced skin injury.

(3) Pain score.

(4) Treatment interruption: Determine whether there is treatment interruption.

(5) Long - term radiation - induced skin injury.

**Table 1. Baseline characteristics of enrolled patients**

| patient ID | age | pathological | stage |  | patient  ID | age | pathological | stage |
| --- | --- | --- | --- | --- | --- | --- | --- | --- |
| 1 | 61 | Squamous cell carcinoma | ⅠB |  | **15** | 51 | Squamous cell carcinoma | Ⅳ |
| 2 | 45 | Small cell neuroendocrine | ⅠB recurrence |  | **16** | 58 | Squamous cell carcinoma | ⅢB |
| 3 | 67 | Squamous cell carcinoma | ⅢA |  | **17** | 43 | Squamous cell carcinoma | ⅢB |
| 4 | 56 | Squamous cell carcinoma | ⅢB |  | **18** | 35 | Squamous cell carcinoma | ⅠA |
| 5 | 60 | Squamous cell carcinoma | ⅠA |  | **19** | 54 | Cervical cancer involving the vulva | Ⅳ |
| 6 | 53 | Squamous cell carcinoma | ⅢA |  | **20** | 53 | Cervical cancer involving the vulva | Ⅳ |
| 7 | 78 | Squamous cell carcinoma | ⅠB recurrence |  | **21** | 68 | Squamous cell carcinoma | ⅢB |
| 8 | 46 | Bartholin’s gland cystadenocarcinoma | Ⅱrecurrence |  | **22** | 52 | Squamous cell carcinoma | ⅠA |
| 9 | 69 | Squamous cell carcinoma | Ⅱrecurrence |  | **23** | 47 | Cervical cancer involving the vulva | Ⅳ |
| 10 | 73 | Squamous cell carcinoma | ⅢA |  | **24** | 54 | Squamous cell carcinoma | ⅣB |
| 11 | 51 | Cervical cancer involving the vulva | ⅢB |  | **25** | 53 | Squamous cell carcinoma | ⅢB |
| 12 | 74 | Squamous cell carcinoma | ⅠB |  | **26** | 67 | Squamous cell carcinoma | ⅠB |
| 13 | 57 | Squamous cell carcinoma | ⅢB |  | **27** | 74 | Squamous cell carcinoma | ⅣA |
| 14 | 52 | Squamous cell carcinoma | ⅢC |  | **28** | 71 | Squamous cell carcinoma | ⅢB |

**prospective cohort studies：**

The study subjects comprised patients with vulvar cancer who were treated at West China Second Hospital of Sichuan University from December 2021 to October 2023. The Ethics Committee of West China Second Hospital of Sichuan University approved the study, and all participants provided informed consent. Inclusion criteria included: female patients aged 18 years or older; patients diagnosed with vulvar malignant tumors, as confirmed by the pathological assessments of two independent pathologists, with no restrictions on the type of pathology; patients requiring radiation therapy that targeted the vulvar region; and study subjects who voluntarily enrolled, signed the informed consent form, demonstrated good compliance, and cooperated with follow-up visits. Exclusion criteria involved patients needing to combine medication due to underlying diseases that could potentially affect the study results, such as the use of oral vascular-targeting drugs.

Patient Admissions and Medications: The first patient was admitted on December 12, 2021. The last patient was admitted on April 29, 2023. Total number of registered cases: 15 (14 surgeries completed).

This study was approved by the West China Second Hospital of Sichuan University (chengdu, China) (ethics approval number: ChiCTR NCT05114226).

**Cell culture and irradiation**

Human embryonic Skin fibroblast HaCaT cells were used as reported previously [1]. The cells were maintained in Dulbecco’s high-glucose modified Eagle medium (HyClone, Logan, UT) supplemented with 10% fetal bovine (Gibco, Grand Island, NY) and incubated at 37°C in a humidified air atmosphere containing 5% CO_2_. Cells were exposed to 10 or 20 Gy of ionizing radiation using an X-ray linear accelerator (KUBTEC XCELL 320, Milford, CT) at a fixed dose rate of 1.7 Gy/min.

**Mice and irradiation**

Protocols for experiments involving mice were approved by the Animal Experimentation Ethics Committee at Sichuan University (Chengdu, China). Control (*GCH1^fl/fl^*; *Krt14*-Cre^-/-^) or *GCH1* conditional KO (*GCH1^fl/fl^*; *Krt14*-Cre^-/-^) male mice were constructed by Cyagen (SuZhou, China). The mice were housed using a 12 h light/dark cycle and had free access to food and water. A model of radiation-induced skin injury in mice by unilateral pulmonary irradiation was established as reported previously [2]. To establish radiogenic injury models, mice were anesthetized with an intraperitoneal injection of pentobarbital sodium (1%, 30 mg/kg), and the hair on hind limb of the mice was shaved using a razor and then immobilized with adhesive tape on a plastic plate to minimize motion during radiation exposure. A 1-cm-thick piece of lead was used to shield the animals (*GCH1^fl/fl^*; *Krt14*-Cre^-/-^ and *GCH1^fl/fl^*; *Krt14*) and localize the radiation field on hind leg. Different doses of irradiation were produced by the accelerator (VARIAN trilogy-sn6518) according to different requirements: 35 Gy at the dose rate of 1000 cGy/min by a 6-MeV electron beam, reactions were followed at regular intervals using the semi-quantitative skin injury scale from 1 (no damage) to 5 (severe damage), as previously described [2].

**Clonogenic survival assay**

HaCaT cells were plated onto 6-well plates. Clonogenic survival assay of cells was performed as reported previously [3]. Then cells were infected with added different concentrations of BH4 for HaCaT cells and irradiated with 0 or 2 Gy of X-ray radiation. Following 10-14 days of incubation, the cells were fixed in methanol, which was followed by crystal violet staining. The number of colonies per well was counted and the survival fractions were calculated as the relative plating efficiencies of the treated cells compared with that of the mock-irradiated (0 Gy) cells. Colonies consisting of 50 or more cells were counted as a clone.

**Western blotting analysis**

Cells were harvested in lysis buffer (BioTeke, Beijing, China) supplemented with 1 mM phenylmethylsulfonyl fluoride and 1 mM protease Inhibitor Cocktail for 20 min. After centrifugation at 4 °C for 10 min (12000 rpm), the supernatant was collected, and protein concentrations were measured using the BCA protein assay kit (Beyotime, Nantong, China). 15-30 μg protein was fractionated by 12.5% SDS-PAGE and electrophoretically transferred to polyvinylidene difluoride (PVDF) membranes (Millipore, Bedford, MA). After blocking with 5% non-fat milk in phosphate-buffered saline (PBS) containing 0.1% Tween-20 (PBST) for 2h at room temperature, the membranes were blotted with primary antibodies PARP (#9532S, Cell Signaling, Beverly, MA) and Tubulin (#2144S, Cell Signaling, Beverly, MA), After washing 3 times with PBST, the membranes were incubated with a horseradish peroxidase (HRP)-conjugated anti-rabbit secondary antibody (#A21020, Abbkine, Wuhan, China) for 2h. Protein bands were visualized and photographed using a FluroChem M imaging system (Shenhua, Hangzhou, China).

**Statistical analysis**

Data are expressed as the mean ± SEM of at least three independent experiments. Multiple treatments were evaluated via one-way ANOVA followed by Tukey’s test for multiple comparisons. Student’s t test was used to compare two groups to determine statistical significance. Survival curves were assessed based on the Kaplan–Meier method and compared using the log-rank test. The statistical analyses were performed using Prism 8 software (GraphPad Software, La Jolla, CA). The differences were considered significant at *P* < 0.05.

**Supplementary Figures and Legends**


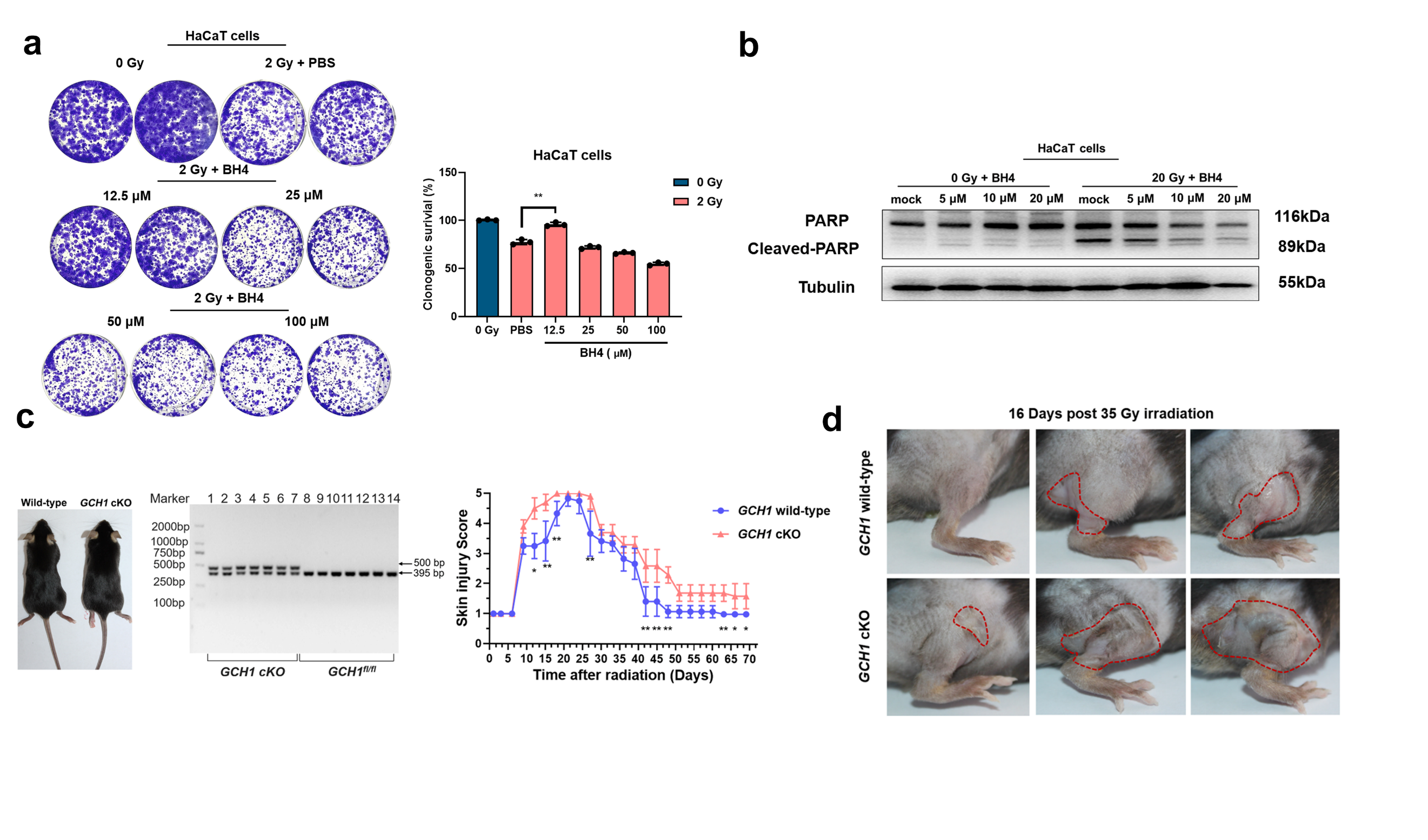


**Supplementary Figure 1.** **The involvement of BH4 in radiation-induced skin injury in vitro and in vivo. (**a) HaCaT cells were treated with BH4 or PBS and subsequently irradiated. The clonogenic survival of the cell lines was measured. (b) Western blotting analyses of PARP expression in irradiated skin cells treated with different BH4 concentrations. **(**c) Typical pictures for *GCH1* (wild-type) and *GCH1* cKO normal mouse were taken, and genotyping was performed using PCR with the primers recommended by Gempharmatech Co., Ltd (Nanjing, China), and the skin tissues from the hind limb region of *GCH1* (wild-type) and *GCH1* cKO mice (n = 5) were irradiation with a single dose of 35 Gy irradiation at a dose rate of 750 cGy/min using a 6-MeV electron beam accelerator. After irradiation, the skin injuries in these groups were measured using a semiquantitative score of 1 (no damage) to 5 (severe damage). (d) Typical pictures of radiogenic skin and claw injury from *GCH1* (wild-type) and *GCH1* (cKO) mice after irradiation. The red arrows indicate skin appendages. The data are presented as mean ± SEM. **P* < 00.5 and ** *P* < 00.1, compared with the control group.

**REFERENCES**

[1]. Qiu Y, Gao Y, Yu D, et al. Genome-Wide Analysis Reveals Zinc Transporter ZIP9 Regulated by DNA Methylation Promotes Radiation-Induced Skin Fibrosis via the TGF-β Signaling Pathway. J Invest Dermatol. 2020,140(1):94-102.e7.

[2]. Song J, Zhang H, Wang Z, et al. The Role of FABP5 in Radiation-Induced Human Skin Fibrosis. Radiat Res. 2018 Feb;189(2):177-186.

[3]. Xie L-W, Cai S, Zhao T-S, et al. Green tea derivative (-)-epigallocatechin-3-gallate (EGCG) confers protection against ionizing radiation-induced intestinal epithelial cell death both in vitro and in vivo. Free Radic Biol Med. 2020,161:175-86.
