# Supplementary figures and images for: Amelioration of radiation-induced skin injury by tetrahydrobiopterin: preclinical study and phase II trial
Source: Mol Biomed. 2025 Jan 26;6:5. doi: 10.1186/s43556-025-00246-x (PMC11762022; doi:10.1186/s43556-025-00246-x)

## The full uncropped Blots images

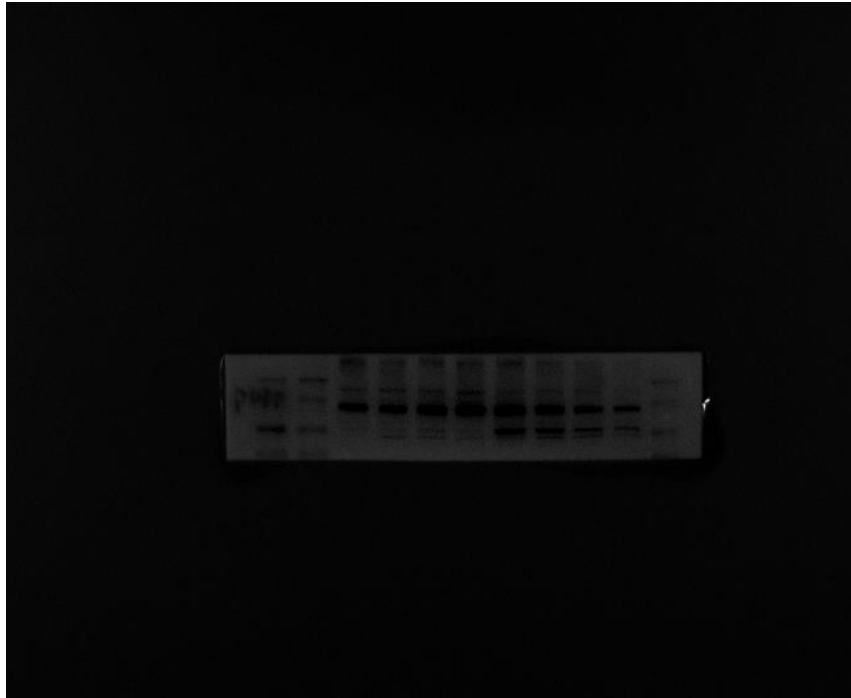

PARP

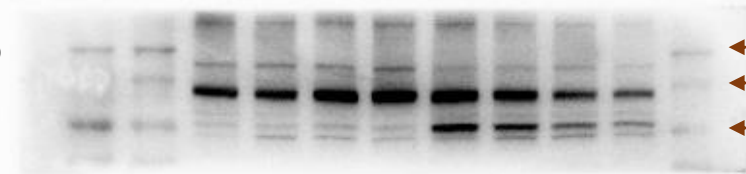

180kDa  
130kDa  
70kDa

## The full uncropped Blots images

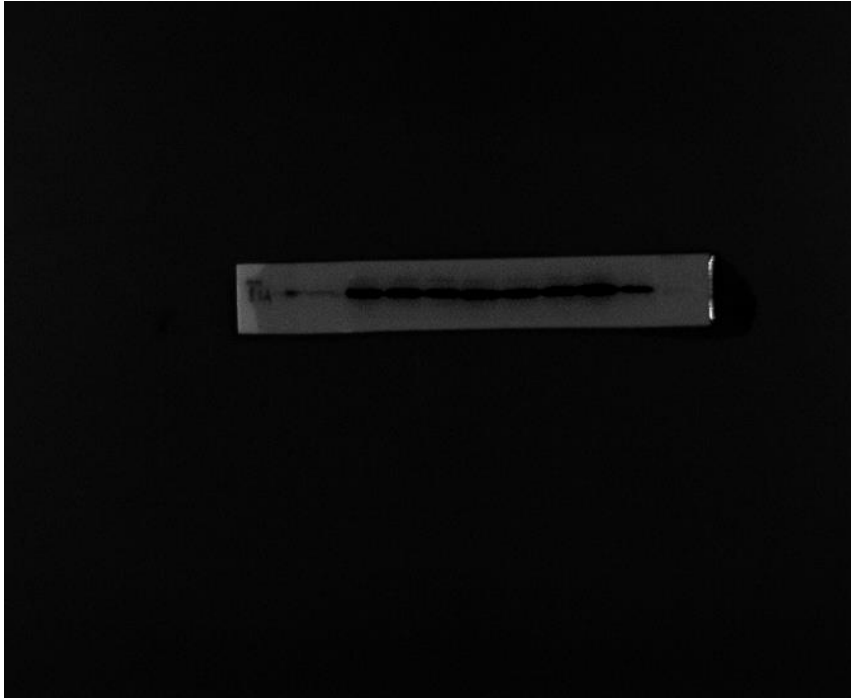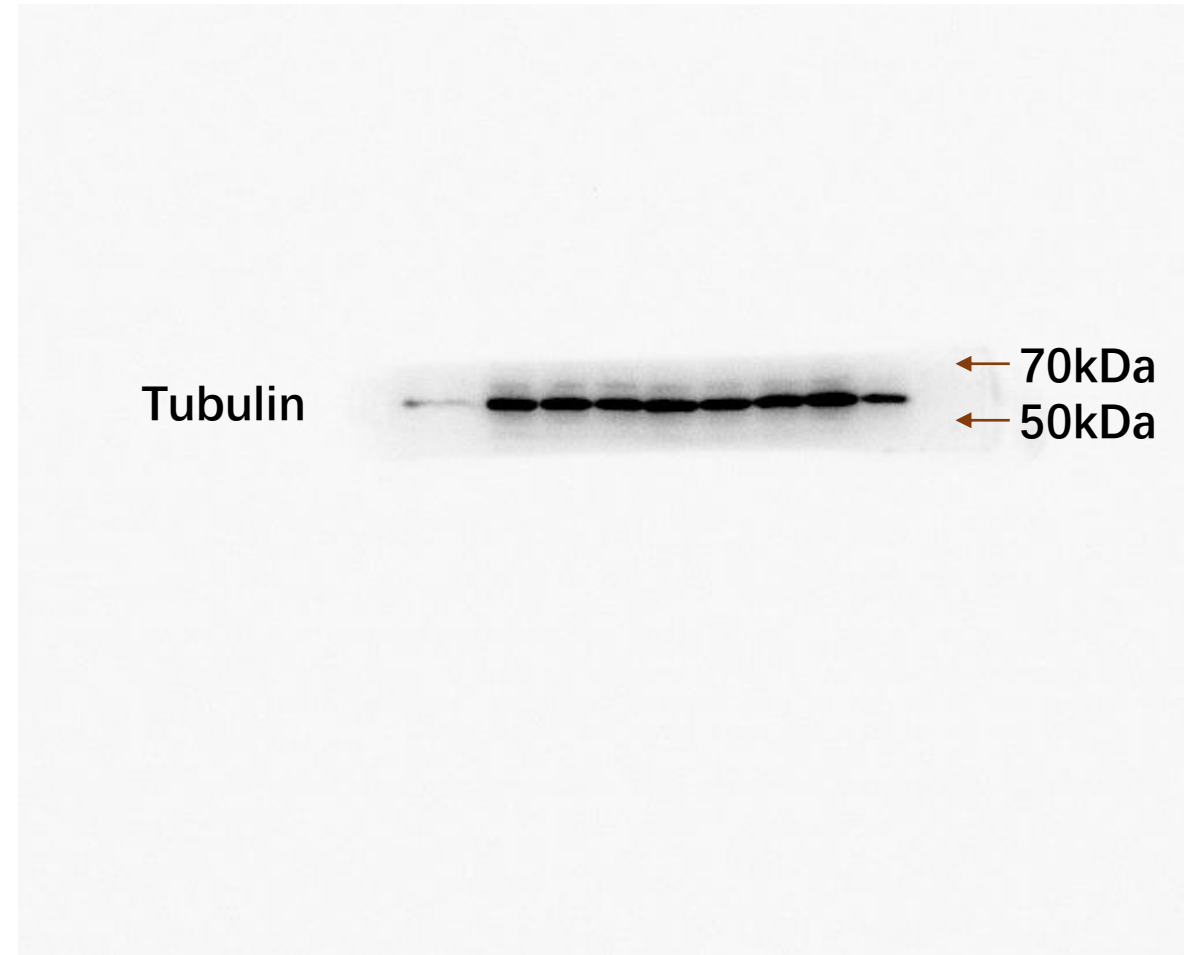

Supplement: Supplementary file 2 — Supplementary Material 2. [file 43556_2025_246_MOESM2_ESM.pdf]

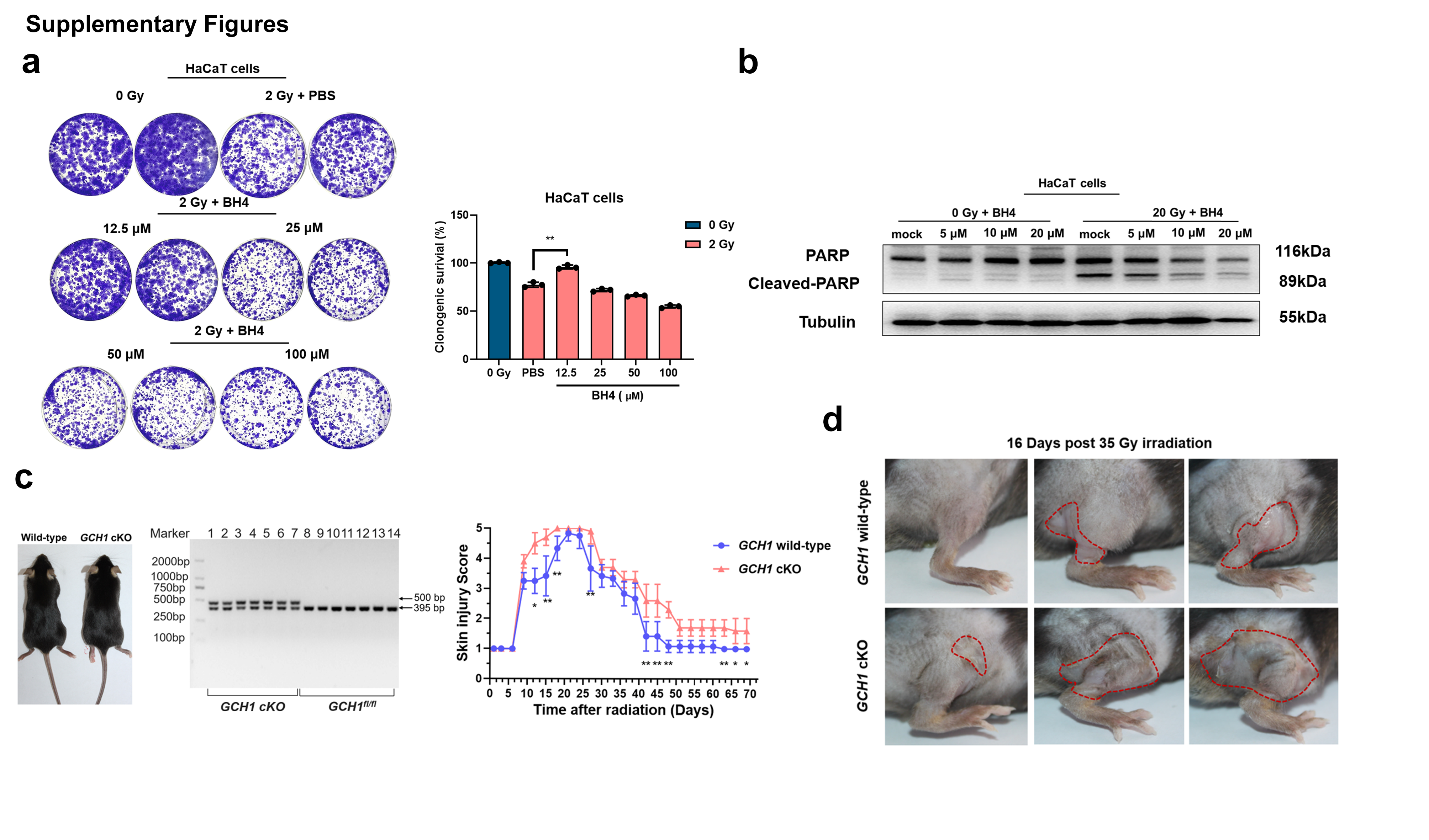

Supplement: Supplementary file 3 — Supplementary Material 3. [file 43556_2025_246_MOESM3_ESM.tif]
